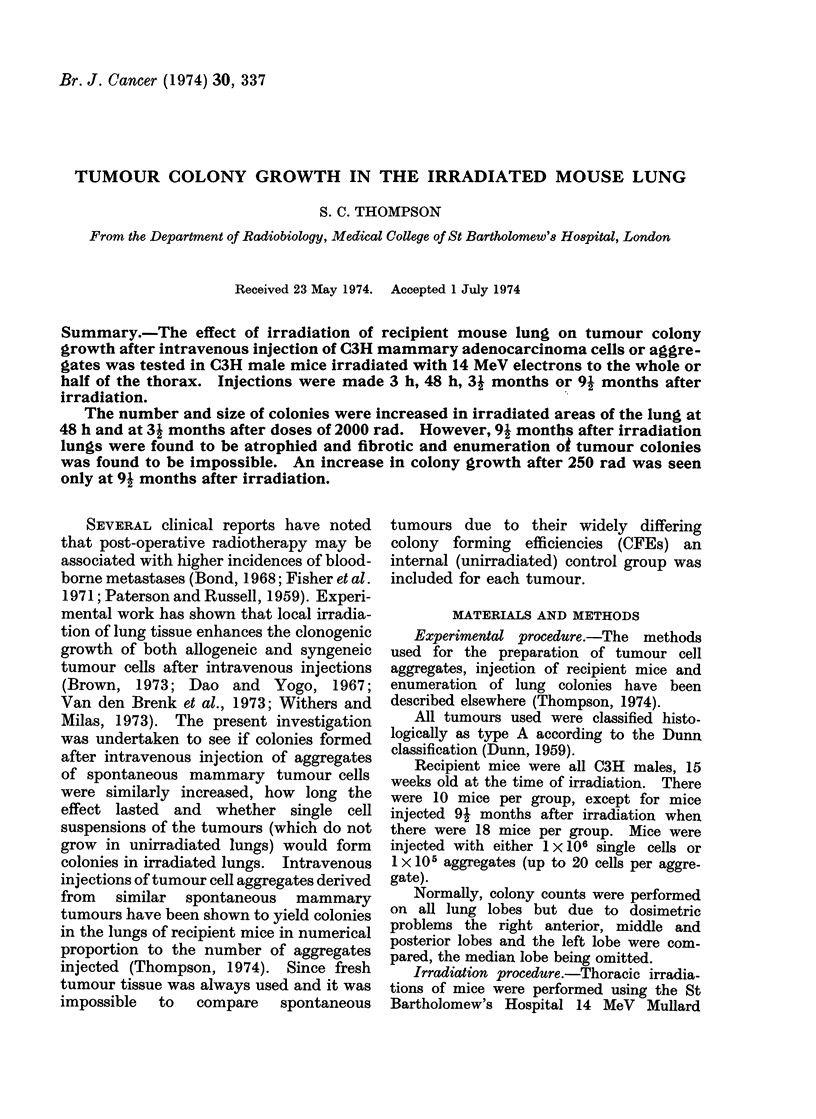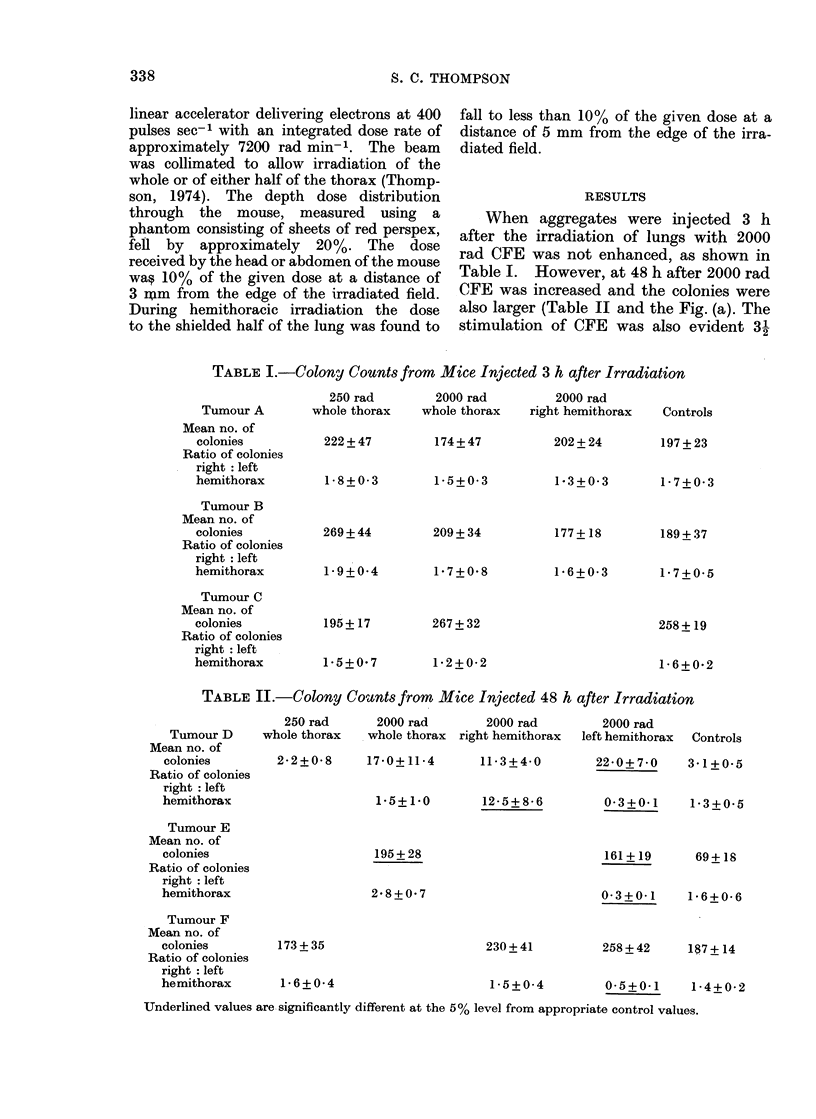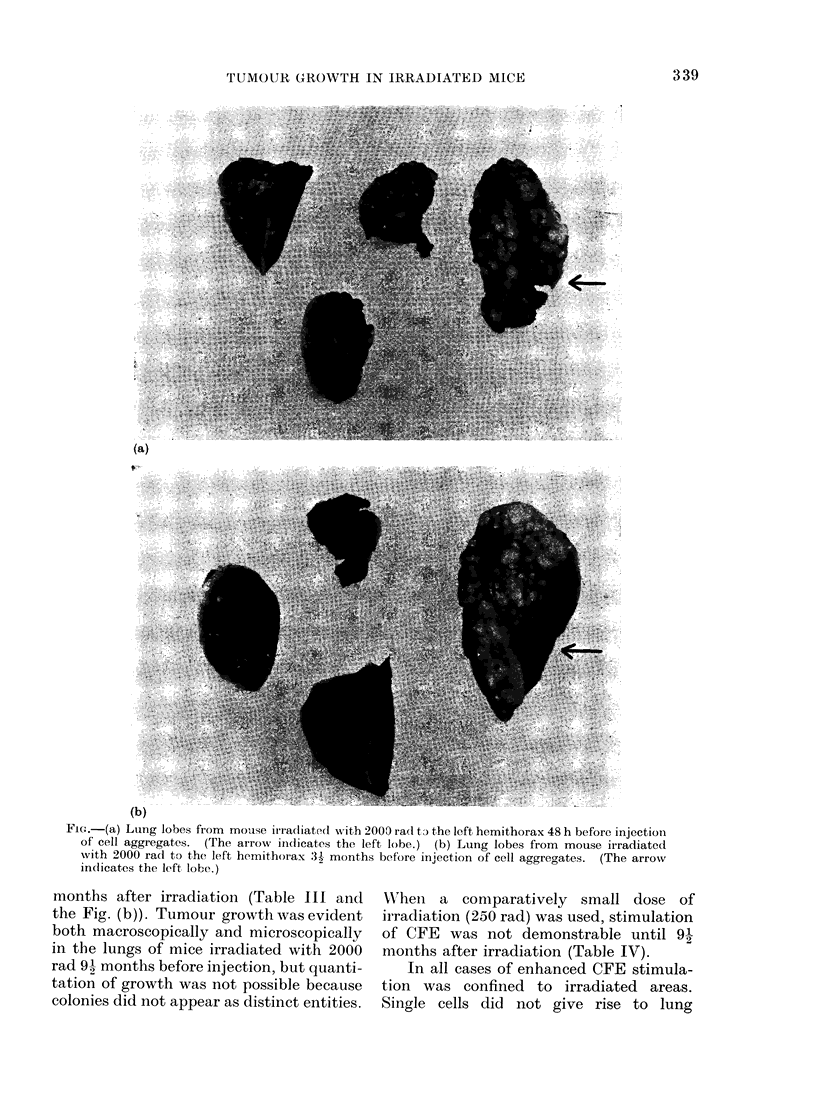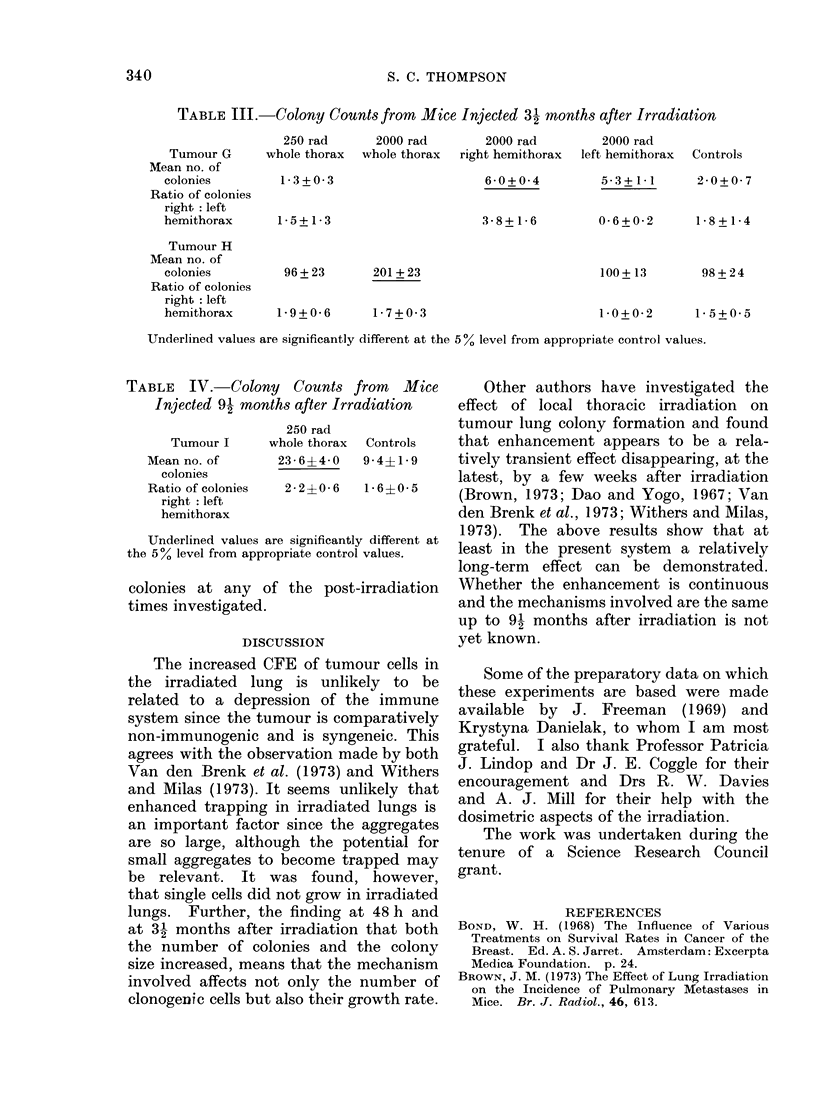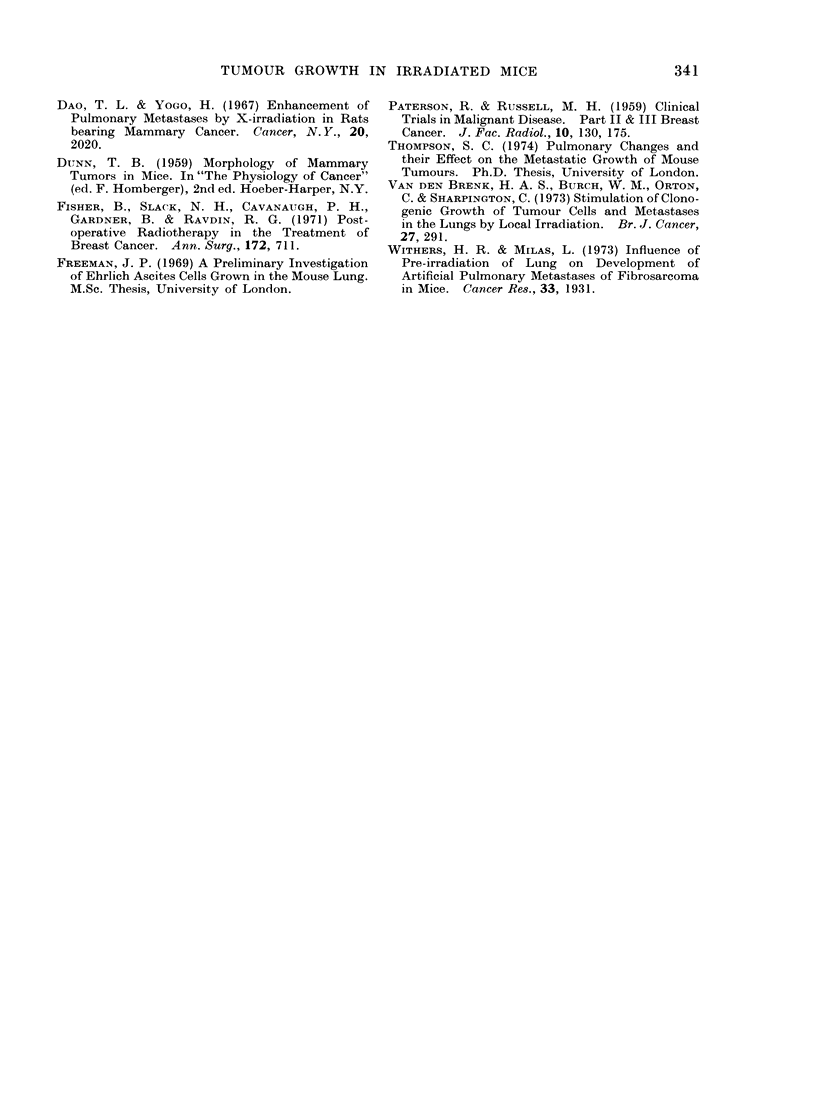# Tumour Colony Growth in the Irradiated Mouse Lung

**DOI:** 10.1038/bjc.1974.202

**Published:** 1974-10

**Authors:** S. C. Thompson

## Abstract

**Images:**


*Br. J. Cancer* (1974) **30**, 337

### TUMOUR COLONY GROWTH IN THE IRRADIATED MOUSE LUNG

S. C. THOMPSON

*From the Department of Radiobiology, Medical College of St Bartholomew's Hospital, London*

Received 23 May 1974. Accepted 1 July 1974

**Summary.—The effect of irradiation of recipient mouse lung on tumour colony growth after intravenous injection of C3H mammary adenocarcinoma cells or aggregates was tested in C3H male mice irradiated with 14 MeV electrons to the whole or half of the thorax. Injections were made 3 h, 48 h, 3½ months or 9½ months after irradiation.**

**The number and size of colonies were increased in irradiated areas of the lung at 48 h and at 3½ months after doses of 2000 rad. However, 9½ months after irradiation lungs were found to be atrophied and fibrotic and enumeration of tumour colonies was found to be impossible. An increase in colony growth after 250 rad was seen only at 9½ months after irradiation.**

SEVERAL clinical reports have noted that post-operative radiotherapy may be associated with higher incidences of blood-borne metastases (Bond, 1968; Fisher *et al*. 1971; Paterson and Russell, 1959). Experimental work has shown that local irradiation of lung tissue enhances the clonogenic growth of both allogeneic and syngeneic tumour cells after intravenous injections (Brown, 1973; Dao and Yogo, 1967; Van den Brenk *et al*., 1973; Withers and Milas, 1973). The present investigation was undertaken to see if colonies formed after intravenous injection of aggregates of spontaneous mammary tumour cells were similarly increased, how long the effect lasted and whether single cell suspensions of the tumours (which do not grow in unirradiated lungs) would form colonies in irradiated lungs. Intravenous injections of tumour cell aggregates derived from similar spontaneous mammary tumours have been shown to yield colonies in the lungs of recipient mice in numerical proportion to the number of aggregates injected (Thompson, 1974). Since fresh tumour tissue was always used and it was impossible to compare spontaneous tumours due to their widely differing colony forming efficiencies (CFEs) an internal (unirradiated) control group was included for each tumour.

### MATERIALS AND METHODS

*Experimental procedure.*—The methods used for the preparation of tumour cell aggregates, injection of recipient mice and enumeration of lung colonies have been described elsewhere (Thompson, 1974).

All tumours used were classified histologically as type A according to the Dunn classification (Dunn, 1959).

Recipient mice were all C3H males, 15 weeks old at the time of irradiation. There were 10 mice per group, except for mice injected 9½ months after irradiation when there were 18 mice per group. Mice were injected with either $1 \times 10^6$ single cells or $1 \times 10^5$ aggregates (up to 20 cells per aggregate).

Normally, colony counts were performed on all lung lobes but due to dosimetric problems the right anterior, middle and posterior lobes and the left lobe were compared, the median lobe being omitted.

*Irradiation procedure.*—Thoracic irradiations of mice were performed using the St Bartholomew's Hospital 14 MeV Mullard linear accelerator delivering electrons at 400 pulses $sec^{-1}$ with an integrated dose rate of approximately 7200 rad $min^{-1}$. The beam was collimated to allow irradiation of the whole or of either half of the thorax (Thompson, 1974). The depth dose distribution through the mouse, measured using a phantom consisting of sheets of red perspex, fell by approximately 20%. The dose received by the head or abdomen of the mouse was 10% of the given dose at a distance of 3 mm from the edge of the irradiated field. During hemithoracic irradiation the dose to the shielded half of the lung was found to fall to less than 10% of the given dose at a distance of 5 mm from the edge of the irradiated field.

### RESULTS

When aggregates were injected 3 h after the irradiation of lungs with 2000 rad CFE was not enhanced, as shown in Table I. However, at 48 h after 2000 rad CFE was increased and the colonies were also larger (Table II and the Fig. (a). The stimulation of CFE was also evident $3\frac{1}{2}$

TABLE I.—*Colony Counts from Mice Injected 3 h after Irradiation*

| | 250 rad whole thorax | 2000 rad whole thorax | 2000 rad right hemithorax | Controls |
|---|---|---|---|---|
| Tumour A | | | | |
| Mean no. of colonies | 222±47 | 174±47 | 202±24 | 197±23 |
| Ratio of colonies right : left hemithorax | 1·8±0·3 | 1·5±0·3 | 1·3±0·3 | 1·7±0·3 |
| Tumour B | | | | |
| Mean no. of colonies | 269±44 | 209±34 | 177±18 | 189±37 |
| Ratio of colonies right : left hemithorax | 1·9±0·4 | 1·7±0·8 | 1·6±0·3 | 1·7±0·5 |
| Tumour C | | | | |
| Mean no. of colonies | 195±17 | 267±32 | | 258±19 |
| Ratio of colonies right : left hemithorax | 1·5±0·7 | 1·2±0·2 | | 1·6±0·2 |

TABLE II.—*Colony Counts from Mice Injected 48 h after Irradiation*

| | 250 rad whole thorax | 2000 rad whole thorax | 2000 rad right hemithorax | 2000 rad left hemithorax | Controls |
|---|---|---|---|---|---|
| Tumour D | | | | | |
| Mean no. of colonies | 2·2±0·8 | 17·0±11·4 | 11·3±4·0 | 22·0±7·0 | 3·1±0·5 |
| Ratio of colonies right : left hemithorax | | 1·5±1·0 | 12·5±8·6 | 0·3±0·1 | 1·3±0·5 |
| Tumour E | | | | | |
| Mean no. of colonies | | 195±28 | | 161±19 | 69±18 |
| Ratio of colonies right : left hemithorax | | 2·8±0·7 | | 0·3±0·1 | 1·6±0·6 |
| Tumour F | | | | | |
| Mean no. of colonies | 173±35 | | 230±41 | 258±42 | 187±14 |
| Ratio of colonies right : left hemithorax | 1·6±0·4 | | 1·5±0·4 | 0·5±0·1 | 1·4±0·2 |

Underlined values are significantly different at the 5% level from appropriate control values.

FIG.—(a) Lung lobes from mouse irradiated with 2000 rad to the left hemithorax 48 h before injection of cell aggregates. (The arrow indicates the left lobe.) (b) Lung lobes from mouse irradiated with 2000 rad to the left hemithorax $3\frac{1}{2}$ months before injection of cell aggregates. (The arrow indicates the left lobe.)

months after irradiation (Table III and the Fig. (b)). Tumour growth was evident both macroscopically and microscopically in the lungs of mice irradiated with 2000 rad $9\frac{1}{2}$ months before injection, but quantitation of growth was not possible because colonies did not appear as distinct entities. When a comparatively small dose of irradiation (250 rad) was used, stimulation of CFE was not demonstrable until $9\frac{1}{2}$ months after irradiation (Table IV).

In all cases of enhanced CFE stimulation was confined to irradiated areas. Single cells did not give rise to lung

TABLE III.—*Colony Counts from Mice Injected $3\frac{1}{2}$ months after Irradiation*

| Tumour G | 250 rad whole thorax | 2000 rad whole thorax | 2000 rad right hemithorax | 2000 rad left hemithorax | Controls |
|---|---|---|---|---|---|
| Mean no. of colonies | $1·3 \pm 0·3$ | | $\underline{6·0 \pm 0·4}$ | $\underline{5·3 \pm 1·1}$ | $2·0 \pm 0·7$ |
| Ratio of colonies right : left hemithorax | $1·5 \pm 1·3$ | | $3·8 \pm 1·6$ | $0·6 \pm 0·2$ | $1·8 \pm 1·4$ |
| Tumour H | | | | | |
| Mean no. of colonies | $96 \pm 23$ | $\underline{201 \pm 23}$ | | $100 \pm 13$ | $98 \pm 24$ |
| Ratio of colonies right : left hemithorax | $1·9 \pm 0·6$ | $1·7 \pm 0·3$ | | $1·0 \pm 0·2$ | $1·5 \pm 0·5$ |

Underlined values are significantly different at the 5% level from appropriate control values.

TABLE IV.—*Colony Counts from Mice Injected $9\frac{1}{2}$ months after Irradiation*

| Tumour I | 250 rad whole thorax | Controls |
|---|---|---|
| Mean no. of colonies | $\underline{23·6 \pm 4·0}$ | $9·4 \pm 1·9$ |
| Ratio of colonies right : left hemithorax | $2·2 \pm 0·6$ | $1·6 \pm 0·5$ |

Underlined values are significantly different at the 5% level from appropriate control values.

colonies at any of the post-irradiation times investigated.

### DISCUSSION

The increased CFE of tumour cells in the irradiated lung is unlikely to be related to a depression of the immune system since the tumour is comparatively non-immunogenic and is syngeneic. This agrees with the observation made by both Van den Brenk *et al.* (1973) and Withers and Milas (1973). It seems unlikely that enhanced trapping in irradiated lungs is an important factor since the aggregates are so large, although the potential for small aggregates to become trapped may be relevant. It was found, however, that single cells did not grow in irradiated lungs. Further, the finding at 48 h and at $3\frac{1}{2}$ months after irradiation that both the number of colonies and the colony size increased, means that the mechanism involved affects not only the number of clonogenic cells but also their growth rate.

Other authors have investigated the effect of local thoracic irradiation on tumour lung colony formation and found that enhancement appears to be a relatively transient effect disappearing, at the latest, by a few weeks after irradiation (Brown, 1973; Dao and Yogo, 1967; Van den Brenk *et al.*, 1973; Withers and Milas, 1973). The above results show that at least in the present system a relatively long-term effect can be demonstrated. Whether the enhancement is continuous and the mechanisms involved are the same up to $9\frac{1}{2}$ months after irradiation is not yet known.

Some of the preparatory data on which these experiments are based were made available by J. Freeman (1969) and Krystyna Danielak, to whom I am most grateful. I also thank Professor Patricia J. Lindop and Dr J. E. Coggle for their encouragement and Drs R. W. Davies and A. J. Mill for their help with the dosimetric aspects of the irradiation.

The work was undertaken during the tenure of a Science Research Council grant.

### REFERENCES

BOND, W. H. (1968) The Influence of Various Treatments on Survival Rates in Cancer of the Breast. Ed. A. S. Jarret. Amsterdam: Excerpta Medica Foundation. p. 24.

BROWN, J. M. (1973) The Effect of Lung Irradiation on the Incidence of Pulmonary Metastases in Mice. *Br. J. Radiol.*, **46**, 613.

DAO, T. L. & YOGO, H. (1967) Enhancement of Pulmonary Metastases by X-irradiation in Rats bearing Mammary Cancer. *Cancer, N.Y.*, **20**, 2020.

DUNN, T. B. (1959) Morphology of Mammary Tumors in Mice. In "The Physiology of Cancer" (ed. F. Homberger), 2nd ed. Hoeber-Harper, N.Y.

FISHER, B., SLACK, N. H., CAVANAUGH, P. H., GARDNER, B. & RAVDIN, R. G. (1971) Post-operative Radiotherapy in the Treatment of Breast Cancer. *Ann. Surg.*, **172**, 711.

FREEMAN, J. P. (1969) A Preliminary Investigation of Ehrlich Ascites Cells Grown in the Mouse Lung. M.Sc. Thesis, University of London.

PATERSON, R. & RUSSELL, M. H. (1959) Clinical Trials in Malignant Disease. Part II & III Breast Cancer. *J. Fac. Radiol.*, **10**, 130, 175.

THOMPSON, S. C. (1974) Pulmonary Changes and their Effect on the Metastatic Growth of Mouse Tumours. Ph.D. Thesis, University of London.

VAN DEN BRENK, H. A. S., BURCH, W. M., ORTON, C. & SHARPINGTON, C. (1973) Stimulation of Clonogenic Growth of Tumour Cells and Metastases in the Lungs by Local Irradiation. *Br. J. Cancer*, **27**, 291.

WITHERS, H. R. & MILAS, L. (1973) Influence of Pre-irradiation of Lung on Development of Artificial Pulmonary Metastases of Fibrosarcoma in Mice. *Cancer Res.*, **33**, 1931.